# Using Visual Guides to Reduce Virtual Reality Sickness in First-Person Shooter Games: Correlation Analysis

**DOI:** 10.2196/18020

**Published:** 2021-07-15

**Authors:** Kwang-Ho Seok, YeolHo Kim, Wookho Son, Yoon Sang Kim

**Affiliations:** 1 BioComputing Lab, Institute for Bio-engineering Application Technology, School of Computer Science and Engineering Korea University of Technology and Education Cheonan-si Republic of Korea; 2 Electronics & Telecommunications Research Institute Daejeon Republic of Korea

**Keywords:** virtual reality, motion sickness, VR sickness, visual guide, VR fidelity

## Abstract

**Background:**

The virtual reality (VR) content market is rapidly growing due to an increased supply of VR devices such as head-mounted displays (HMDs), whereas VR sickness (reported to occur while experiencing VR) remains an unsolved problem. The most widely used method of reducing VR sickness is the use of a rest frame that stabilizes the user's viewpoint by providing fixed visual stimuli in VR content (including video). However, the earth-fixed grid and natural independent visual background that are widely used as rest frames cannot maintain VR fidelity, as they reduce the immersion and the presence of the user. A visual guide is a visual element (eg, a crosshair of first-person shooter [FPS]) that induces a user's gaze movement within the VR content while maintaining VR fidelity, whereas there are no studies on the correlation of visual guide with VR sickness.

**Objective:**

This study aimed to analyze the correlation between VR sickness and crosshair, which is widely used as a visual guide in FPS games.

**Methods:**

Eight experimental scenarios were designed and evaluated, including having the visual guide on/off, the game controller on/off, and varying the size and position of the visual guide to determine the effect of visual guide on VR sickness.

**Results:**

The results showed that VR sickness significantly decreased when visual guide was applied in an FPS game. In addition, VR sickness was lower when the visual guide was adjusted to 30% of the aspect ratio and positioned in the head-tracking direction.

**Conclusions:**

The experimental results of this study indicate that the visual guide can achieve VR sickness reduction while maintaining user presence and immersion in the virtual environment. In other words, the use of a visual guide is expected to solve the existing limitation of distributing various types of content due to VR sickness.

## Introduction

Recently, virtual reality (VR) content based on head-mounted display (HMD) has been expanded to various industrial fields such as sports, medical care, education, and social network. Moreover, such content has been used in video games. However, most users have experienced “VR sickness” while using HMD-based VR content. VR sickness has symptoms similar to motion sickness, including nausea, oculomotor discomfort, and disorientation caused while experiencing an HMD-based VR [1]. To investigate the causes of VR sickness, various theories are being studied in a cognitive science approach. The popular sensory conflict theory [1] proposes that VR sickness is induced by an inconsistency between the visual and the vestibular or proprioceptive senses. In particular, the vection that occurs during the VR experience is the biggest cause of sensory conflict [2-4]. Additionally, the postural instability theory posits that VR sickness is caused by changes in human balance [5]. Furthermore, VR sickness may also be induced by various individual characteristics, such as age, gender, prior user experience, concentration, medical history, mental rotation, perceptual style, and dominant eye [6].

Until now, various studies have only been partially successful in attempting to reduce HMD-induced VR sickness by focusing on the device and the content. A typical method focusing on the device is the optimization of the delay time caused by head movement tracking response, rendering, image transmission, and display response speed [7]. Studies have also been conducted to reduce VR sickness in device elements such as resolution, frame rate, viewing angle, binocular parallax, and flicker fusion frequency [8-13]. Recent studies have evaluated methods focusing on content. These methods investigated the use of dynamic blurring with retinal tracking [14], optical flow reduction of peripheral vision [15], field of view control [16], and viewpoint snapping [17].

However, there is a disadvantage that these dynamic blurring methods limit the user's experience. Therefore, in other studies, VR sickness has been reduced by adding fixed or dynamic visual stimuli regardless of the motion of objects in the content [18-20]. This visual stimulus includes a rest frame that serves as a reference frame designed to induce a user's effective spatial perception. In particular, VR sickness was reduced by applying a virtual human nose as a rest frame to the content [21]. However, this artificial rest frame could not maintain VR fidelity, as it reduced user presence and immersion and provided a strong sense of heterogeneity to the user. VR sickness has further been reduced using the earth-fixed grid or the natural independent visual background (IVB) with rest frames, whereas it did not increase the presence and immersion of the user [22,23]. Particularly, VR sickness in first-person shooter (FPS) games was reduced when cockpits were added to the IVB, while the VR fidelity of the users was disturbed [24].

To overcome the problem of IVB, we discuss another visual element applied to VR FPS games called the *visual guide*, which refers to a visual element that induces a user's gaze movement within VR content. To our knowledge, there is no study investigating the effect of visual guide on the reduction of VR sickness while maintaining VR fidelity in VR FPS games. Therefore, in this study, we investigated how VR sickness reduction is affected by visual guide in FPS games. To do this, we performed experiments on a VR FPS game consisting of eight scenarios, including visual guide on/off, game controller on/off, and varying the size and position of the visual guide.

## Methods

### Visual Guide Design in VR FPS Games

**Figure 1:**
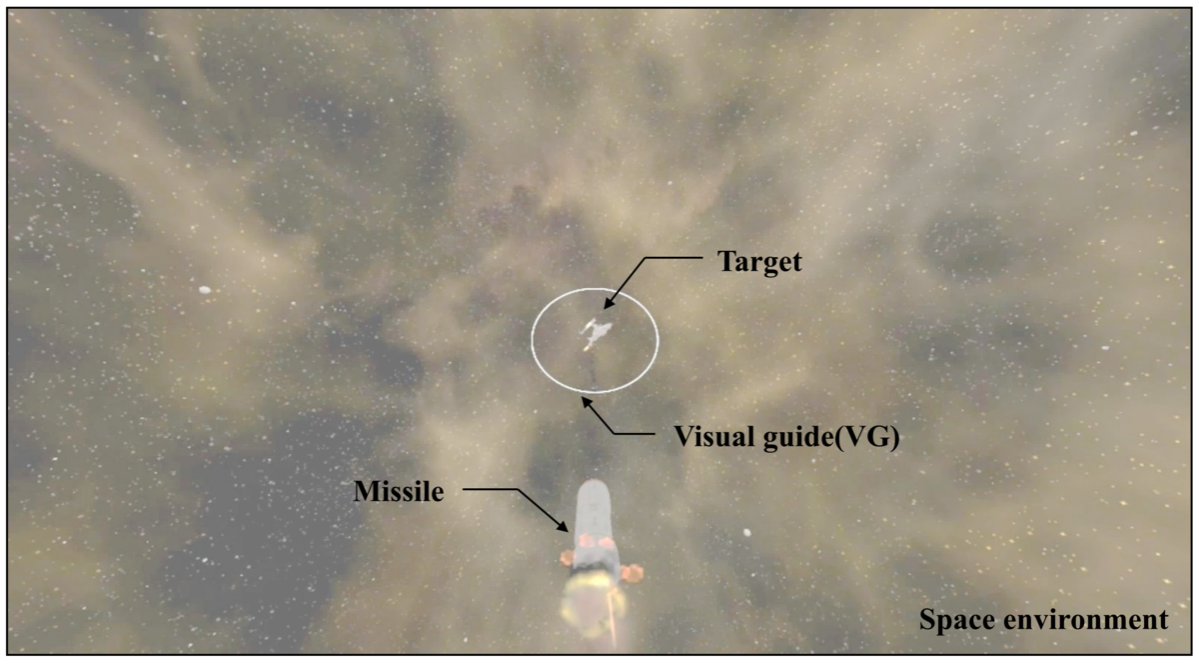
Example of crosshair used in the virtual reality first-person shooter game. HMD: head-mounted display; SSQ: Simulator Sickness Questionnaire.

In this section, we describe the visual guide used in this study. In VR FPS games, crosshairs, character path indicators, and maps are used as visual guides to provide situational awareness for the user to spatially determine their position in a virtual environment. In this study, we used crosshair as a visual guide to induce a user's gaze movement while maintaining VR fidelity in a space-environment FPS game. Crosshair is a 2D image composed of color, shape, line thickness, depth, size, and position elements. Figure 1 shows an example of crosshair used in the VR FPS game. The brilliant color, complex shape, bold line, and depth of a visual guide can reduce the presence and immersion of the user by increasing the visual stimulation [18]. Therefore, these elements are fixed, and they are designed as a white, circle, 1.0 px, and 0, respectively by referring to commercial VR FPS games. Table 1 shows various crosshair in commercial VR FPS games.

**Table 1:** Various crosshair in commercial virtual reality (VR) first-person shooter (FPS) games.

No.	Name	Crosshair image
1	Super Stardust Ultra VR	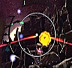
2	End Space VR	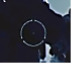
3	Elite: Dangerous	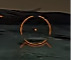
4	Gunjack	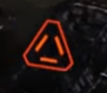
5	Sublevel Zero	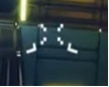
6	VR Galaxy Wars	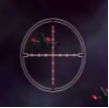
7	Fusion Wars	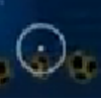
8	EVE: Valkyrie	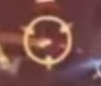
9	Rez Infinite	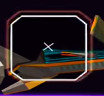
10	EVERSPACE	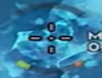

The size and position of the visual guide is considered a variable for VR sickness measurements. Therefore, we designed it as a variable. First, the size was designed to be 0%, 10%, 30%, and 50% of the size of the aspect ratio (with 0% implying that there is no visual guide). Second, the position was synchronized to “none,” “head-tracking direction with HMD (H),” “movement direction with game controller (G),” and “head-tracking direction with HMD and movement direction with game controller (H & G).” That is, if the position is “none,” then there is no visual guide. If the position is “H,” then the position of the visual guide is synchronized with the head-tracking direction by the HMD operation. If the position is “G,” then the position of the visual guide is synchronized in the direction of forwarding movement, pitch, yaw, and roll rotation by the game controller operation. Finally, if the position is “H & G,” then the position of the visual guide is synchronized with the head-tracking direction by the HMD operation and the direction of forwarding movement, pitch, yaw, and roll by the game controller operation.

### Experiments

In this section, we describe the experimental environment and methods used to examine the effect of visual guide on VR sickness. The experimental environment consisted of the participant, experiment device, and experimental content.

We used a paired *t* test method to analyze the results. This requires the experimental results to approximate the normal distribution. Therefore, the minimum sample size was set to 30 to satisfy a central limit theorem [25]. Eventually, 32 individuals (male: 23, female: 9) participated in the experiment.

All the participants were in their twenties without any HMD VR experience, and none had a medical history of hearing or balancing disorder. We received an institutional review board approval for testing of VR sickness by KOREATECH.

Before performing the experiment, each participant was administered a preliminary questionnaire (M1) comprising the Simulator sickness Questionnaire (SSQ) items, such as measurement, reliability, validity, score interpretation, etc [26,27], to evaluate their current motion sickness state. After the SSQ (M1) was completed, the participant wore the HMD, and the experiment was initiated.

The experimental equipment used was a head-tracking interface device HTC VIVE HMD and an Xbox One Wireless Controller. With these devices, the participants played the VR FPS game and answered the SSQ. The game controller has several functionalities including forward movement; rotation based on pitch, yaw and roll axes; missile launch targeting; and selection of answers in the SSQ. Experimental content is a VR FPS game in space environment because it easily causes VR sickness due to multiaxis movement. The user uses the HMD and the game controller operations to trace the enemy target and score it with the missile.

**Figure 2:**
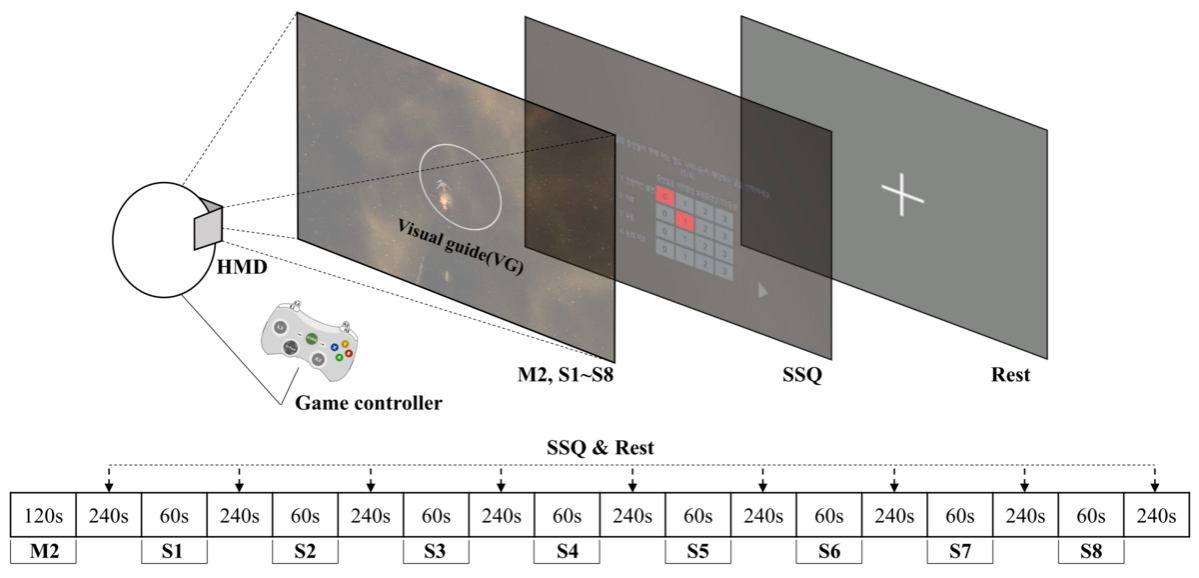
Experimental protocol for virtual reality sickness measurement. HMD: head-mounted display; SSQ: Simulator Sickness Questionnaire.

The experimental protocol consisted of preliminary experiment (M2), eight experimental scenarios (S1–S8), SSQ, and rest videos. Figure 2 shows the experimental protocol for VR sickness measurement. In the preliminary experiment (M2), the participants were taught how to operate the HMD and the game controller. The eight experimental scenarios were designed to measure VR sickness. If a participant is exposed to VR content for a long time, the VR sickness level will potentially increase. [28] Therefore, each scenario was composed of content of 60 s for the safety of the participants. These eight experimental scenarios were designed to have the visual guide on/off, the game controller on/off, and to vary sizes and positions of the visual guide to determine its effect on VR sickness. Table 2 shows the features of these eight scenarios.

In addition, the experimental scenarios were randomly placed and used to ensure reliability. For 240 s after each experimental scenario (including M2) ended, participants entered SSQ and watched the rest video to relax VR sickness. All participants were equally exposed to this rest video. When the experiment was completed, the SSQ data for the eight scenarios were automatically saved. From the VR sickness measurement experiment, preexperimental SSQ data (M1) and postexperimental SSQ data (S1-S8) were collected for each participant.

**Table 2:** Features of eight scenarios used in the experiment.

Feature	S1	S2	S3	S4	S5	S6	S7	S8
Game controller	Off	On	On	On	On	On	On	On
Visual guide size (%)	0	0	0	10	30	50	30	30
Visual guide position	None	None	None	H^a^	H	H	G^b^	H & G^c^

^a^H: head-tracking direction with head-mounted display.

^b^G: movement direction with game controller.

^c^H & G: head-tracking direction with head-mounted display and movement direction with game controller.

**Figure 3:**
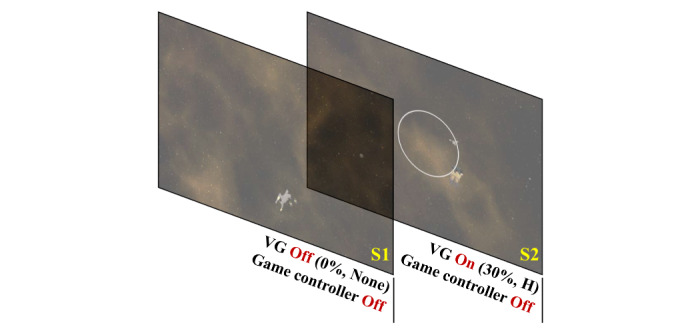
S1 and S2 used in the first step of experiment I. VG: visual guide.

Using these experimental protocols, we conducted two experiments to examine the effect of the visual guide on VR sickness. Experiment I investigated the effect of visual guide on/off on VR sickness. In the first step of experiment I, we investigated the effect of VR sickness when the visual guide is on/off with the game controller off. Thus, the SSQ data of S1 and S2 were used for the comparison of VR sickness. Figure 3 shows the S1 and S2 used in the first step of experiment I.

**Figure 4:**
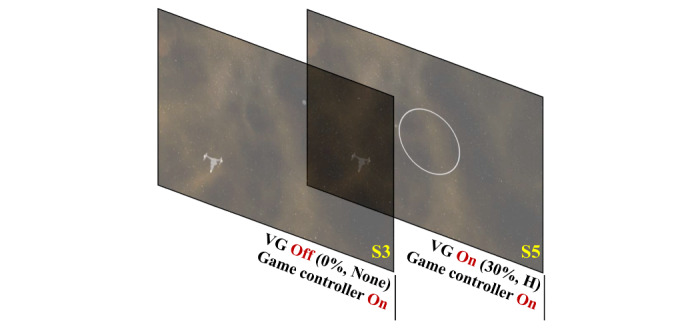
S3 and S5 used in the second step of experiment I. VG: visual guide.

In the second step of experiment I, we investigated the effect of visual guide on/off with the game controller on. Thus, the SSQ data of S3 and S5 were used for the comparison of VR sickness. Figure 4 shows the S3 and S5 used in the second step of experiment I.

**Figure 5:**
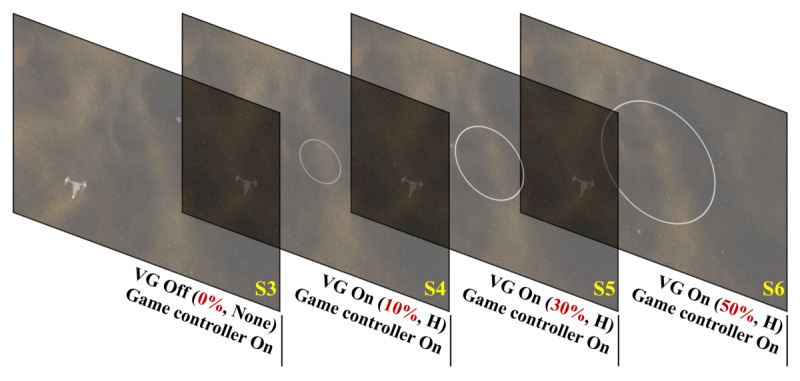
S3, S4, S5, and S6 used in the first step of experiment II. VG: visual guide.

Experiment II investigated the effects of the visual guide’s size and position on VR sickness. In the first step of experiment II, we investigated the effect of the visual guide’s size with the game controller on. Thus, the SSQ data of S4, S5, and S6 and the SSQ data of S3 were used for the comparison of VR sickness. Figure 5 shows the S3, S4, S5, and S6 used in the first step of experiment II.

**Figure 6:**
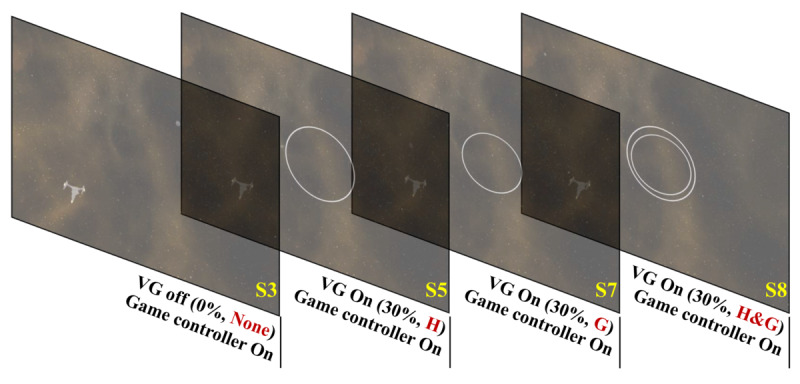
S3, S5, S7, and S8 used in the second step of Experiment II. VG: visual guide.

In the second step of experiment II, we investigated the effect of the visual guide’s position with the game controller on. Thus, the SSQ data of S5, S7, and S8 using a visual guide with different positions at 30% of the size of the aspect ratio and the SSQ data of S3 were used for comparison of VR sickness. Figure 6 shows the shows S3, S5, S7, and S8 used in the second step of experiment II.

## Results

### Overview

First, we compared the SSQ preexperiment (M1) and postexperiment (S1-S8) data to verify that the experimental protocol for VR sickness measurement was well designed. From the paired *t* test results, a significant difference was observed in VR sickness between M1 and S1-S8. Significantly higher nausea and disorientation symptoms were noted in S1-S8 than in M1. Hence, we confirmed that the participants had experienced nausea and disorientation. In addition, oculomotor discomfort in S1-S8 did not significantly increase because many participants had eye fatigue from M1. From the different SSQ values before and after VR exposure, the increase in the values of nausea and disorientation symptoms of the participants was confirmed. It was concluded that the scenarios for the VR sickness experiment were appropriately produced.

### Experiment I: Effects of Visual Guide On/Off

**Figure 7:**
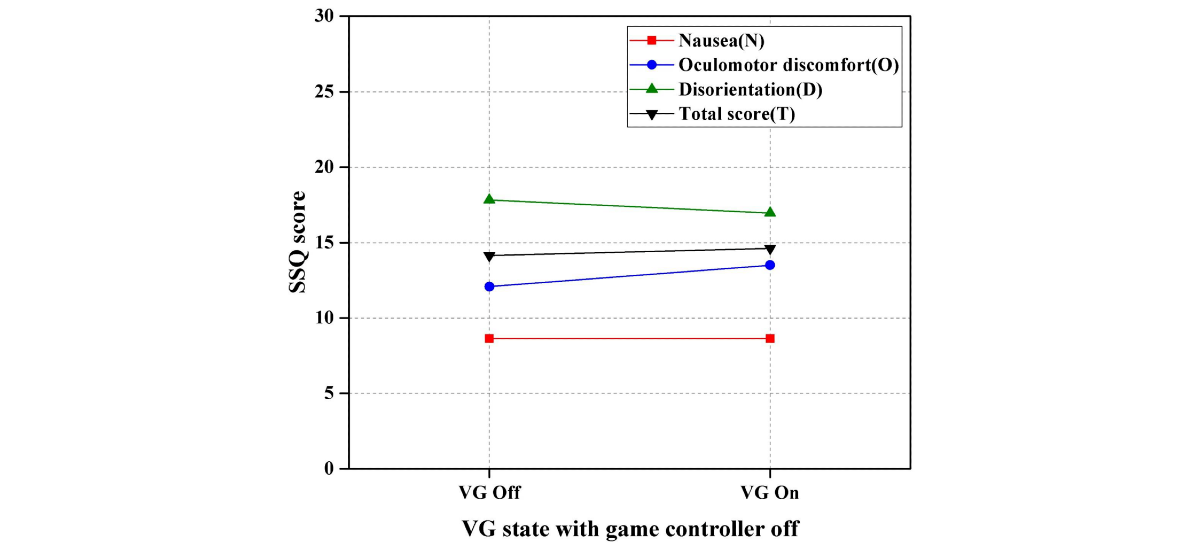
Results of the first step of experiment I (difference in virtual reality sickness with respect to visual guide on/off with game controller off). SSQ: Simulator Sickness Questionnaire; VG: visual guide.

In the first step of experiment I, the results of the paired *t* test showed no significant difference in VR sickness with respect to visual guide on/off when game controller was off; there was a negligible difference in nausea, oculomotor discomfort, disorientation, and total score values. In other words, there was no effect of visual guide with game controller off (watching video) on VR sickness. Table 3 shows the results of the paired *t* test of visual guide on/off with game controller off. Figure 7 shows the results of the first step of experiment I (ie, difference in VR sickness with respect to visual guide on/off with game controller off).

**Figure 8:**
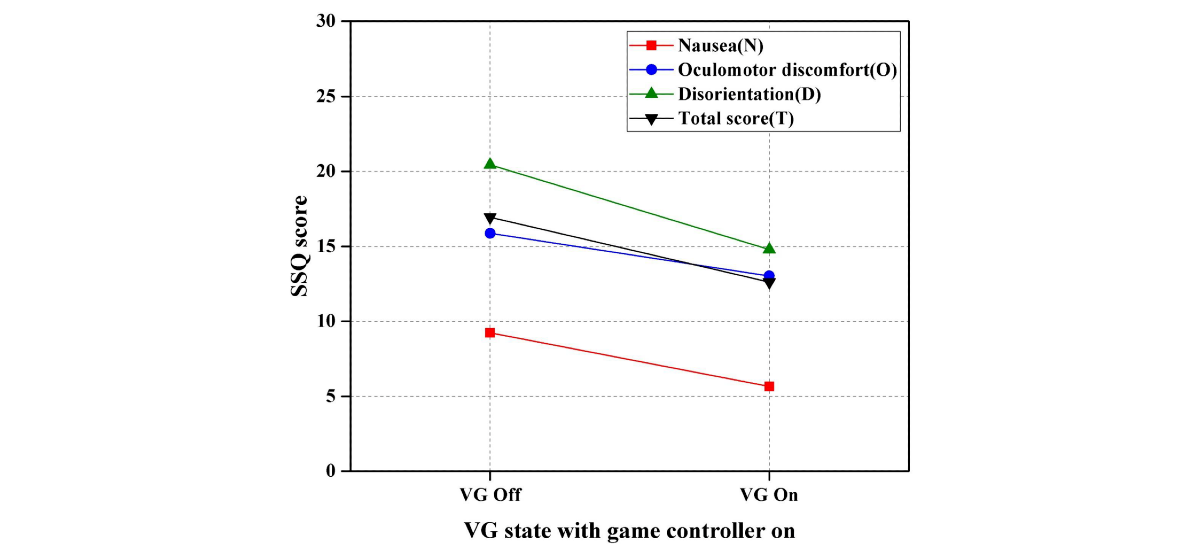
Results of the second step of experiment I (difference in virtual reality sickness of visual guide on/off with game controller on). SSQ: Simulator Sickness Questionnaire; VG: visual guide.

In the second step of Experiment I, the paired *t* test showed a significant difference in VR sickness with visual guide on/off and game controller on: nausea and total score values were significantly decreased, whereas oculomotor discomfort and disorientation values had no significant differences. Table 4 shows the results of the paired *t* test of visual guide on/off with the game controller on. Figure 8 shows the results of the second step of Experiment I (difference in VR sickness of visual guide on/off with game controller on).

**Table 3:** Results of the paired *t* test of the visual guide on/off with game controller off.

Scenario (state)	Simulator Sickness Questionnaire														
	Nausea				Oculomotor discomfort				Disorientation				Total score		
	Score	*t* (*df*)	P value	Score		*t* (*df*)	P value	Score		*t* (*df*)	P value	Score		*t* (*df*)	P value
S1 (visual guide off)	8.65	N/A^a^	N/A	12.08		N/A	N/A	17.84		N/A	N/A	14.14		N/A	N/A
S2 (visual guide on)	8.65	0 (31)	>.99	13.50		–.649 (31)	.52	16.97		.360 (31)	.72	14.61		–.235 (31)	.82

^a^N/A: not applicable.

**Table 4:** Results of the paired *t* test of the visual guide on/off with game controller on.

Scenario (state)	Simulator Sickness Questionnaire														
	Nausea				Oculomotor discomfort				Disorientation				Total score		
	Score	*t* (*df*)	P value	Score		*t* (*df*)	P value	Score		*t* (*df*)	P value	Score		*t* (*df*)	P value
S3 (visual guide off)	9.24	N/A^a^	N/A		15.87	N/A	N/A		20.45	N/A	N/A		16.95	N/A	N/A
S5 (visual guide on)	5.66	2.547 (31)	*.02* ^b^		13.03	1.359 (31)	.18		14.79	1.815 (31)	.08		12.62	2.183 (31)	*.04*

^a^N/A: not applicable.

^b^Italicized values indicate statistical significance.

### Experiment II: Effect of Visual Guide Size and Position

**Figure 9:**
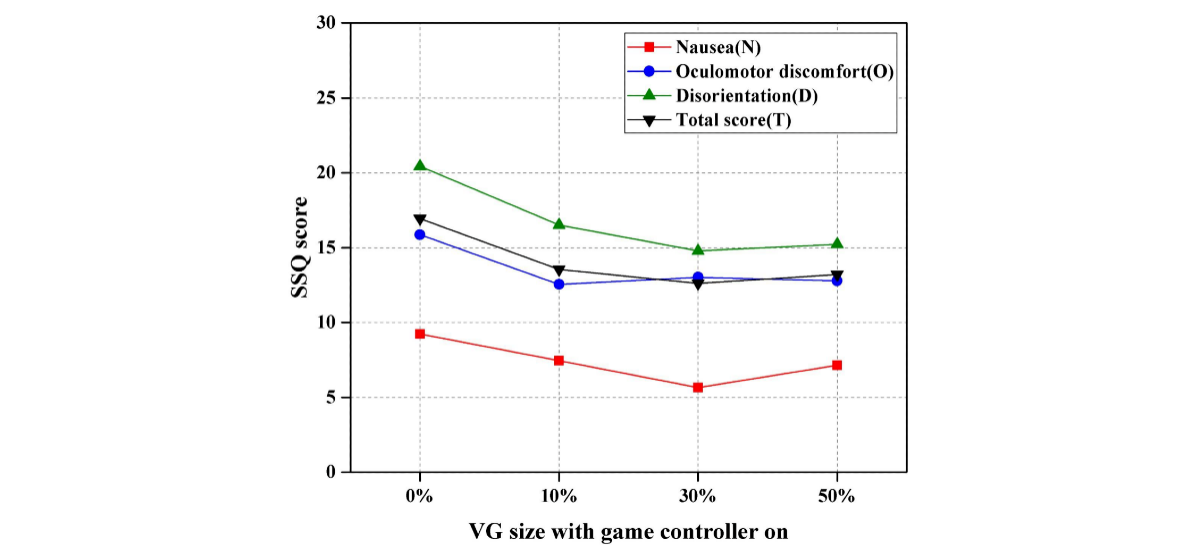
Results of the first step of experiment II (difference in virtual reality sickness with respect to visual guide size when game controller was on). SSQ: Simulator Sickness Questionnaire; VG: visual guide.

In the first step of Experiment II, the paired *t* test showed that there was a significant difference in VR sickness with the size of visual guide used in S5: nausea and total score values were significantly decreased, whereas oculomotor discomfort and disorientation values had no significant differences. However, there was no significant difference in VR sickness with the size of visual guide used in S4 and S6. Table 5 shows the results of the paired *t* test of the visual guide size with the game controller on. As shown in Figure 9, VR sickness was lower than that observed with other sizes when the visual guide was 30% of the size of the aspect ratio. Figure 9 shows the results of the first step of Experiment II (ie, difference in VR sickness with respect to the visual guide size when the game controller was on).

**Figure 10:**
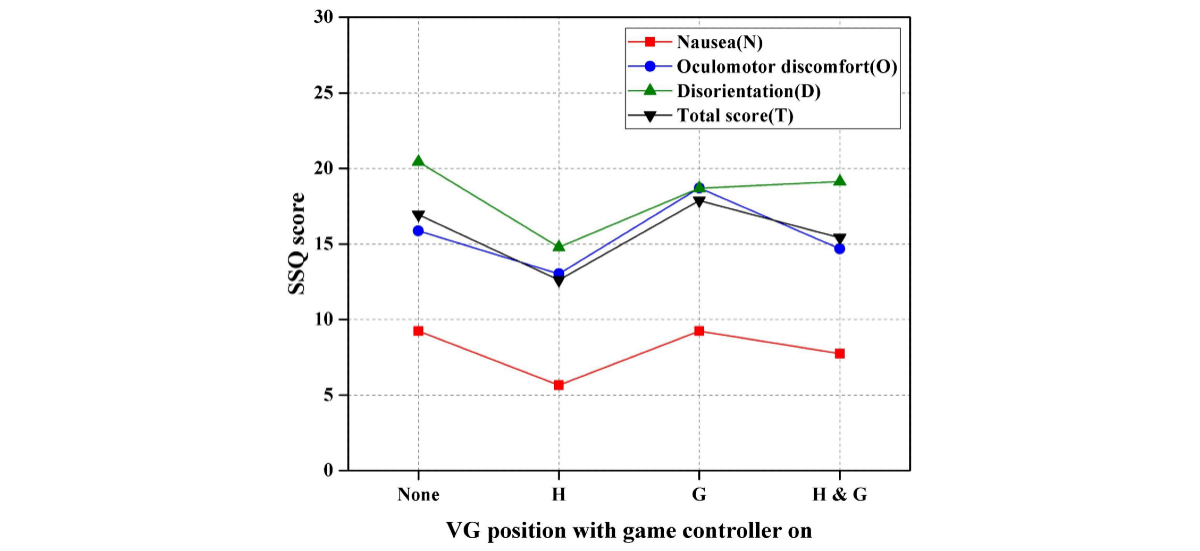
Results of the second step of Experiment II (difference in virtual reality sickness with respect to visual guide position when game controller is on). SSQ: Simulator Sickness Questionnaire; VG: visual guide.

In the second step of Experiment II, the paired *t* test showed that there was a significant difference in VR sickness with the position of visual guide used in S5: nausea and total score values were significantly decreased, whereas oculomotor discomfort and disorientation values had no significant differences. However, there was no significant difference in VR sickness with the position of the visual guide used in S7 and S8. Table 6 shows the results of the paired *t* test of the visual guide position with the game controller on. As shown in Figure 10, VR sickness was lower than that observed with other positions when visual guide was in the head-tracking direction. Figure 10 shows the results of the second step of Experiment II (ie, difference in VR sickness with respect to visual guide position when the game controller is on).

**Table 5:** Results of the paired *t* test of visual guide size with game controller on.

Scenario (size)	Simulator Sickness Questionnaire														
	Nausea				Oculomotor discomfort				Disorientation				Total score		
	Score	*t* (*df*)	P value	Score		*t* (*df*)	P value	Score		*t* (*df*)	P value	Score		*t* (*df*)	P value
S3 (0%)	9.24	N/A^a^	N/A		15.87	N/A	N/A		20.45	N/A	N/A		16.95	N/A	N/A
S4 (10%)	7.45	1.063 (31)	.296		12.55	1.422 (31)	.17		16.53	1.359 (31)	.18		13.56	1.674 (31)	.10
S5 (30%)	5.66	2.547 (31)	*.02* ^b^		13.03	1.359 (31)	.18		14.79	1.815 (31)	.08		12.62	2.183 (31)	*.04*
S6 (50%)	7.16	0.980 (31)	.34		12.79	1.200 (31)	.24		15.23	1.615 (31)	.12		13.21	1.447 (31)	.16

^a^N/A: not applicable.

^b^Italicized values indicate statistical significance.

**Table 6:** Results of the paired *t* test of the visual guide position with game controller on.

Scenario (position)	Simulator Sickness Questionnaire													
	Nausea			Oculomotor discomfort				Disorientation				Total score		
	Score	*t* (*df*)	P value	Score	*t* (*df*)	P value	Score		*t* (*df*)	P value	Score		*t* (*df*)	P value
S3 (none)	9.24	N/A^a^	N/A	15.87	N/A	N/A	20.45		N/A	N/A	16.95		N/A	N/A
S5 (H)	5.66	2.547 (31)	*.02* ^b^	13.03	1.359 (31)	18	14.79		1.815 (31)	.08	12.62		2.183 (31)	*.04*
S7 (G)	9.24	0 (31)	>.99	18.71	–1.099 (31)	.28	18.71		0.436 (31)	.67	17.88		–0.323 (31)	.75
S8 (H & G)	7.75	0.645 (31)	.52	14.69	0.456 (31)	.65	19.14		0.317 (31)	.75	15.43		0.517 (31)	.61

^a^N/A: not applicable.

^b^Italicized values indicate statistical significance.

## Discussion

### Principal Findings

In this study, we analyzed the correlation between VR sickness and crosshair, which is widely used as a visual guide in an FPS game. To do this, eight scenarios were designed: visual guide on/off, game controller on/off, and various sizes and positions of visual guide. Experiments were performed using a protocol that consisted of the abovementioned eight scenarios, SSQ, and rest video. Results of Experiment I showed no reduction of VR sickness of the visual guide when the game controller was not operated, whereas there was a reduction when the game controller was operated. While the user operated the HMD and the game controller in the game, the user's gaze movement was synchronized with the motion of the visual guide, thereby reducing VR sickness. The results suggest that visual guide can reduce VR sickness caused by sensory conflicts between content and users when manipulating content. It can be interpreted that visual guide should be used effectively to reduce VR sickness because most VR content requires a game controller. Results of experiment II confirmed a difference in VR sickness with respect to the size and position of the visual guide with game controller operation. VR sickness according to sizes 10% and 50% and positions G and H & G of the visual guide was not significant, whereas VR sickness according to size 30% and position H of the visual guide was significant. Moreover, VR sickness was lower when the visual guide was 30% of the size of the aspect ratio and positioned in the head-tracking direction compared with other sizes and positions. The various sizes of the visual guide reduced nausea, oculomotor discomfort, disorientation symptom, and total score when compared to scenarios when a visual guide was not used. In particular, the visual guide at 30% the size of the aspect ratio further reduced these symptoms to a larger extent than other sizes. The visual guide at 50% of the size of the aspect ratio reduced the disorientation symptom, showing that disorientation was minimized due to the high synergy with the gaze movement when the visual guide size increased. However, if the size of the visual guide increases, the VR fidelity cannot be maintained because it lowers the immersion and presence of the user. The visual guide positioned in the head-tracking direction reduced the symptoms of nausea, oculomotor discomfort, disorientation, and total score more than other positions. As mentioned above, crosshair was the visual guide function to reduce VR sickness, as well as maintain VR fidelity in FPS games.

When recruiting participants, we tried to keep the number of men and women the same. As a result, the number of male (n=23) participants exceeded the number of female (n=9) participants recruited. This may have affected the results of the experiments, as it is reported that men are stronger than women with regard to motion sickness [29]. Therefore, the effects of the proposed method in a same-gender ratio environment should be included in further study.

### Conclusions

In this study, we used an experimental protocol consisting of scenarios such as visual guide on/off, game controller on/off, and various sizes and positions of the visual guide to analyze the correlation between VR sickness and crosshair that is widely used as a visual guide in FPS games. VR sickness was found to be significantly correlated with visual guide on/off, and the use of a visual guide was very effective in reducing VR sickness when using a game controller. VR sickness was reduced by synchronizing the user's gaze movement to the motion of the visual guide while operating the HMD and game controller within the game. In addition, VR sickness reduced when the visual guide was 30% of the size of the aspect ratio and positioned in the head-tracking direction. From these findings, it is confirmed that using a visual guide can be an effective method to reduce VR sickness. The experimental results of this study indicate that the visual guide can achieve VR sickness reduction while maintaining user presence and immersion in the virtual environment. In other words, the use of visual guide is expected to solve the existing difficulty in disseminating various VR content due to VR sickness.

## Abbreviations

FPS: first-person shooter
G: movement direction with game controller
H: head-tracking direction with head-mounted display
H & G: head-tracking direction with head-mounted display and movement direction with game controller
HMD: head-mounted display
IITP: Institute for Information & Communications Technology Promotion
IVB: independent visual background
MSIT: Ministry of Science and ICT
SSQ: Simulator Sickness Questionnaire
VR: virtual reality
